# A 5-step root cause analysis model for test overutilization: A study on its application to plasma transferrin testing

**DOI:** 10.1093/ajcp/aqae015

**Published:** 2024-03-06

**Authors:** Jiracha Jittapranerat, Wimol Chinswangwatanakul

**Affiliations:** Department of Clinical Pathology, Faculty of Medicine Siriraj Hospital, Mahidol University, Nakhon Pathom, Thailand; Department of Clinical Pathology, Faculty of Medicine Siriraj Hospital, Mahidol University, Nakhon Pathom, Thailand

**Keywords:** laboratory utilization management, root cause analysis, test overutilization, transferrin

## Abstract

**Objectives:**

This study aimed to develop a root cause analysis (RCA) model for test overutilization, applying it to transferrin overordering at our institution.

**Methods:**

A comprehensive review was undertaken to establish a systematic RCA model. Upon implementation, the questionnaire identifying the root causes of transferrin overordering with infographic intervention was distributed to clinicians and nurses.

**Results:**

The RCA model comprises 5 steps: (1) problem identification, (2) causal factor determination, (3) data collection, (4) significant factor identification, and (5) corrective action development and outcome measurement. The major causes of transferrin overutilization were confusion between transferrin and transferrin saturation, as well as unfamiliarity with the laboratory handbook. An infographic reduced postintervention transferrin ordering among clinicians (84.9%, *P* < .001) and nurses (46.8%, *P* < .001).

**Conclusions:**

This study presents a 5-step RCA model that offers a customized method to identify the causes of test overutilization. Applying this model to transferrin at our institution revealed 22 leading root causes. Laboratories are encouraged to adopt this RCA model as it can contribute to optimized patient care and more efficient resource allocation.

**KEY POINTS**

- Root cause analysis (RCA) is fundamental to addressing laboratory overutilization.
- Our proposed RCA model, consisting of 5 reproducible steps, offers a comprehensive approach applicable to the investigation of overused tests.
- Corrective action delivered through a knowledge infographic demonstrated desirable outcomes.

## INTRODUCTION

Laboratory test overutilization is characterized by the ordering of tests for which the results are either irrelevant or not beneficial to patient care.^1,2^ A meta-analysis discovered that overutilization constituted approximately 20% of all tests.^2^ This issue poses a significant challenge in medicine as it can lead to additional costs and indirectly influences patient care, such as patient anxiety, unnecessary blood collection, competition with the necessary test, and misdiagnosis.^2-6^ From a laboratory perspective, this problem is called a “pre-preanalytical laboratory error,” and it is the most prevalent type of laboratory error in real-world practice.^7,8^

Comparable to other laboratories, the central laboratory of the Department of Clinical Pathology at the Faculty of Medicine Siriraj Hospital encounters test overutilization issues, one of which involves plasma transferrin. A recent study demonstrated that plasma transferrin does not provide supplementary information when co-ordered with iron studies (serum iron, total iron-binding capacity) and ferritin tests in anemic patients.^9^ Furthermore, transferrin levels exhibit low accuracy in diagnosing and treating iron deficiency anemia.^10,11^ As a result, plasma transferrin is considered redundant and overused in the context of iron deficiency anemia.

The growing awareness of laboratory test overuse within the health care community has led to the development of numerous methods to address this issue. In the context of quality improvement management, these methods are referred to as laboratory utilization management (LUM) or demand management.^1,3-5,12^ Although the primary principle of LUM centers on conducting laboratory tests on the right patients, at the right times, and for the right reasons, LUM also encompasses other strategies and diverse outcomes.^12,13^ Consequently, a systematic approach to overutilization is necessary to achieve better outcomes before implementing LUM.^1,3^

To promote effective laboratory test utilization, it is essential to understand the root causes of overordering.^1,3^ Initial studies identified several reasons for diagnostic test overuse, including physicians’ lack of knowledge, patients’ demands, medicolegal factors, and the multitude of diagnostic tests that are available for doctors to prescribe.^14-16^ In addition, focus group interviews revealed 3 primary factors contributing to general overutilization: personal, organizational, and technical.^17^ However, successful LUM implementation not only requires the identification of causal factors for test overutilization but also demands the exploration of the root causes of each issue before the implementation.^1,3^ Therefore, if a root cause analysis (RCA) is not conducted, this remaining gap may contribute to ineffective LUM in certain situations.

Implementing an RCA uncovers a comprehensive understanding of the underlying factors contributing to a specific problem.^3^ This is particularly valuable in the context of medical errors, where RCA proves its effectiveness in identifying and addressing the root causes of incidents, ultimately enhancing patient safety.^18^ In the laboratory situation, the RCA is often used in quality improvement projects that involve nonconformity analysis, turnaround time reduction, or patient identification issue.^19^ Several tools of RCAs have successfully been applied for those quality improvement conditions such as causal factor chart, fishbone diagram, Pareto chart, and driver diagram.^1,3,19^

At our institution, a noticeable increase in plasma transferrin overordering was observed in 2020. Laboratory data indicated a 2-fold rise in plasma transferrin orders over two fiscal years (2019-2020). A retrospective chart review revealed that orders were placed using various methods, including clinicians writing the orders themselves and nurses selecting the orders on a request form. An RCA was initially considered to determine the reasons for the overordering. While RCA undoubtedly offers valuable insights for optimizing laboratory operations.^1,3^ its complexity seems to be a significant barrier to its implementation.^18,19^ Hence, RCA remains infrequently employed in laboratory settings, especially in test misordering. Despite the inherent challenges, RCA of plasma transferrin overuse is essential to address the gap and enhance the effectiveness of LUM. Consequently, we aimed to develop a tailored approach to RCA that better suits our specific laboratory context. This study’s objectives were (1) to establish the RCA model for identifying significant root causes of laboratory test overordering and (2) to implement this model for plasma transferrin tests as an LUM strategy to reduce overutilization.

## METHODS

This study consisted of 2 parts: (1) the development of the RCA model and (2) the application of the proposed model to plasma transferrin ordering at our institution.

### Developing the RCA Model

To develop a customized RCA model, we extensively reviewed publications on RCA protocols in laboratory medicine^3,19^ and collected protocols from both national^20^ and international sources.^3,18,19,21^

### Applying the RCA Model to Plasma Transferrin Ordering

Upon establishing a 5-step RCA model, we applied it to plasma transferrin ordering at our institution. Per the model’s third step, we conducted a survey to identify the root causes of plasma transferrin overutilization. Two separate questionnaires (Supplemental Data; all supplementary material is available at *American Journal of Clinical Pathology* online) were designed for physicians and nurses based on their typical practices and responsibilities at our institution. Both questionnaires consisted of 5 sections: demographic data, pretest ordering behavior evaluation, a knowledge infographic, posttest evaluation, and self-evaluation for RCA of transferrin ordering. The self-evaluation part for each group incorporated root causes from an Ishikawa diagram (Fishbone diagram), and specific root causes for both occupations were assessed separately.

Cochran’s formula (N = Z^2^pq/e^2^) was primarily used to estimate the sample size, accounting for 25% incomplete data. The final sample size included 173 respondents per group. Our initial attempt at random sampling yielded an insufficient response rate, prompting us to adopt convenience, snowball, and quota sampling techniques. This approach increased accessibility and expanded our sample size to encompass a more diverse group of doctors and nurses. The data collection was conducted from August 2021 to September 2022. Before this research began, its protocol was approved by Siriraj’s Institutional Review Board (Si-621/2021).

Statistical analyses were performed using IBM SPSS Statistics for Windows, version 27, employing descriptive and analytic statistics. Proportions in percentages were used for descriptive categorical data. The χ^2^ and McNemar tests were applied to test the hypothesis for single-time and pre-post testing, respectively. Probability (*P*) values lower than .05 indicated statistical significance.

## RESULTS

### Developing the RCA Model

Following extensive research, the simple and systematic 5-step RCA model Table 1 was developed.

**TABLE 1:** Five Steps of the Root Cause Analysis Model for Laboratory Test Overutilization

Step of root cause analysis	Tool
1. Describe the adverse event and ordering process	Process flow diagram
2. Identify root causes and causal factors	Fishbone diagram
3. Collect information	Questionnaire
4. Determine important factors	Pareto chart
5. Develop corrective actions and measure the outcomes	Plan-do-study-act cycle

#### Description of Adverse Event and the Process

This initial step involved reviewing clinical documentation and asking, “What/when/who/how/why did the event happen?”^18,20^ A process flowchart aided in understanding the sequence of actions, involved personnel, and critical steps leading to the adverse event.^18,21^

#### Root Cause Identification and Causal Factor Charting

An Ishikawa diagram (Fishbone diagram) was employed to visualize all potential reasons contributing to the problem.^3^ The major groups of causes were used to systematically initiate the main branches: manpower, machines, materials, and methods. Deeper causes were explored and documented within the main branches.^3,21^ The causes in our fishbone chart were derived from the authors’ brainstorming, retrospective chart review, and previous study with similar conditions.

#### Data Collection and Questionnaire Development

This step’s primary objective was to conduct interviews and brainstorming with all parties involved in all possible causes.^18^ Due to the lack of a utilization committee, organizing a multidisciplinary team-brainstorming session was challenging.^13,19^ Consequently, we designed questionnaires for mass distribution to physicians and nurses involved in the ordering process.

#### Significant Root Cause Determination

A Pareto chart was a bar graph illustrating the Pareto principle, which posited that 80% of events result from 20% of causes. The chart arranged bars in descending order of frequency, with a line graph representing the cumulative percentage of the total superimposed on the bars.^21,22^ This chart quantified causes and identified a few crucial root causes for intervention targeting.^3,21^ Our Pareto chart was created from the frequency of responses for each root cause described in the questionnaire.

#### Corrective Project Execution and Outcome Measurement

The RCA results were futile without corrective action.^19^ Implementing a small trial test with a plan-do-study-act (PDSA) cycle was vital for change effectiveness.^3^ Plan: We planned to correct misunderstandings causing overutilization. Do: Educational infographics were incorporated into physicians’ and nurses’ questionnaires. Study: Outcomes were studied using pre-post testing to ensure appropriate implementation. Act: Further modification strategies were considered for improvement.

### Applying the RCA Model to Plasma Transferrin Ordering

#### Flowchart of Laboratory Test Ordering

**FIGURE 1:**
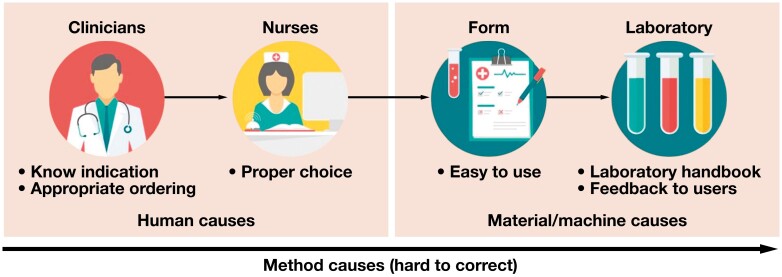
The top-down flowchart of the laboratory test-ordering process (images from Flaticon.com).

The problem of overordering plasma transferrin measurements in iron deficiency anemia was illustrated using a flowchart. The flowchart depicted the sequential order of people, materials, and machines in our institution’s routine laboratory ordering pathway Figure 1. Clinicians appropriately document laboratory tests with proper indications in patient charts. Subsequently, nurses select the ordered tests on request forms (using traditional paper forms at outpatient clinics and electronic devices in inpatient settings). Our user-friendly request forms allow easy test selection with a simple tick. Last, the laboratory performs the ordered tests. Laboratory communication about test utilization includes a laboratory handbook on our institution’s website and direct feedback to clinicians and nurses. Other significant and difficult-to-correct potential causes are method elements such as order-writing protocols for physicians, the large number of laboratories and request forms in our institution, and the difficulty of testing cancellation after the inappropriate order was detected.

#### Fishbone Diagram of Transferrin Ordering Causes

**FIGURE 2:**
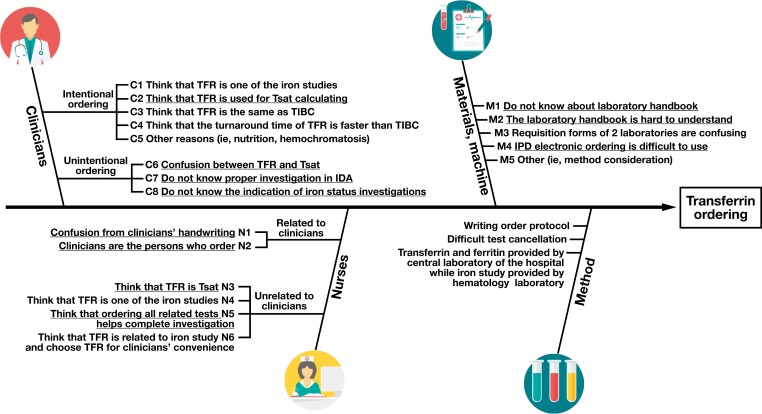
The Ishikawa (fishbone) diagram of the transferrin ordering process (images from Flaticon.com). The bold-underlined causes are significant from the Pareto analysis. IDA, iron deficiency anemia; IPD, inpatient department; TIBC, total iron-binding capacity; Tsat, transferrin saturation.

The fishbone diagram Figure 2 identified 22 root causes, categorized into 4 groups: clinicians, nurses, materials and machines, and methods. The 19 causes falling in the first 3 groups were coded for questionnaire purposes. Clinician causes were split into intentional ordering (C1-C5) and unintentional ordering (C6-C8), while nurse-ordering factors were divided into clinician-related (N1-N2) and unrelated (N3-N6) categories. The materials and machines group contained 5 specific causes (M1-M5). However, the 3 causes within the methods group remained uncoded due to the difficulty in addressing these factors at our institution.

#### Data Collection: Demographic Profiles and Ordering Behavior of Respondents

Following the creation of the fishbone diagram for plasma transferrin ordering, questionnaires were distributed to clinicians and nurses. A total of 101 clinicians (58.4% of the estimated sample size) and 165 nurses (95.4% of the estimated sample size) responded to the questionnaires. The majority of the respondents were female, and most of the clinicians and nurses were from the Department of Internal Medicine. Residents constituted the majority of respondents in the clinician group, while inpatient nurses had the highest response rate among nursing subgroups Table 2.

**TABLE 2:** Demographic Data of the Participants

Characteristic	Clinicians (n = 101), No. (%)	Nurses (n = 165), No. (%)
Sex		
Male	40 (39.6)	3 (1.8)
Female	60 (59.4)	159 (96.4)
Not disclosed	1 (1)	3 (1.8)
Specialist		
Internal medicine	29 (28.7)	59 (35.8)
Pediatric	16 (15.8)	46 (27.9)
Gynecology and obstetric	13 (12.9)	1 (0.6)
Surgery	11 (10.9)	14 (8.5)
Orthopedic	7 (6.9)	20 (12.1)
Emergency	6 (5.9)	14 (8.5)
Other (psychiatry, anesthesiology, etc)	19 (18.8)	11 (6.7)
Clinician’s position		
Resident	84 (83.2)	NA
Fellow	7 (6.9)	NA
Staff	10 (9.9)	NA
Nurse’s working unit		
Inpatient	NA	107 (64.8)
Outpatient	NA	44 (26.7)
Emergency room	NA	14 (8.5)

NA, not applicable.

The pretest evaluation examined the ordering behaviors of clinicians and nurses. Clinicians ordered plasma transferrin tests for iron deficiency anemia by writing the test in the patient chart for nurse ordering (7.92%) or by choosing the test in the request forms themselves (32.7%). There was a significant difference between the writing and choice selecting protocol (*P* < .001). Surprisingly, all transferrin orders by clinicians were selected simultaneously with iron studies and ferritin tests. Similar to the results with the clinician group, a pretest questionnaire revealed similar inappropriate transferrin ordering behavior among nurses. While nurses typically select tests based on clinician orders, a concerning 75.3% of them independently chose plasma transferrin in the pretest without clinician input.

#### Pareto Chart for Identifying Significant Causes

**FIGURE 3:**
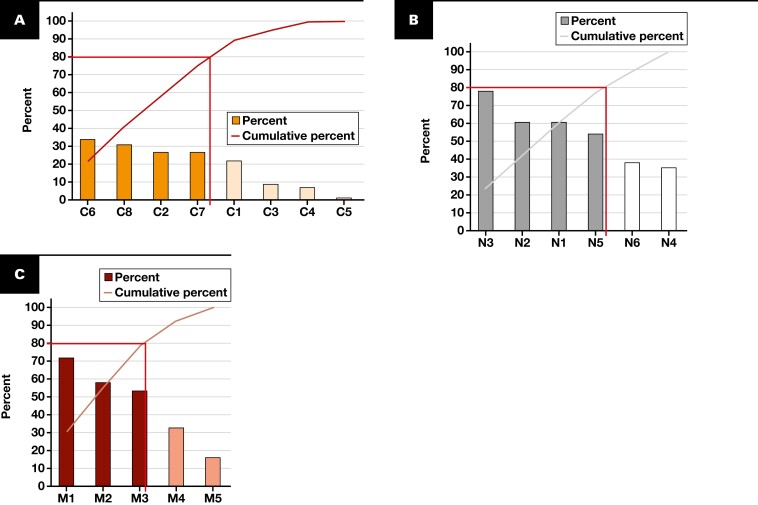
The Pareto charts for the transferrin ordering process. **A**, Clinician causes. **B**, Nurse causes. **C**, Material and machine causes.

The fishbone diagram Figure 2 illustrates the significant causes of transferrin ordering, as determined from Pareto charts Figure 3. The vital root causes among clinicians were confusion between transferrin and transferrin saturation (C6), lack of knowledge about iron study indications (C8), misunderstanding that transferrin is used to calculate transferrin saturation (C2), and lack of knowledge about investigations for iron deficiency anemia (C7). The critical reasons among nurses were confusion between transferrin and transferrin saturation (N3), clinician orders (N2), poor clinician handwriting (N1), and ordering all related tests to ensure a complete investigation (N5). Finally, issues with the laboratory handbook (M1, M2) were mostly found in the materials and methods group, followed by difficulty using inpatient electronic ordering (M3). Other causes of overordering were hemochromatosis diagnosis, pretransfusion protocol, hematologist consultation, and inadequate education and communication (C5, M5).

#### Laboratory Utilization Management Intervention and Evaluation

**FIGURE 4:**
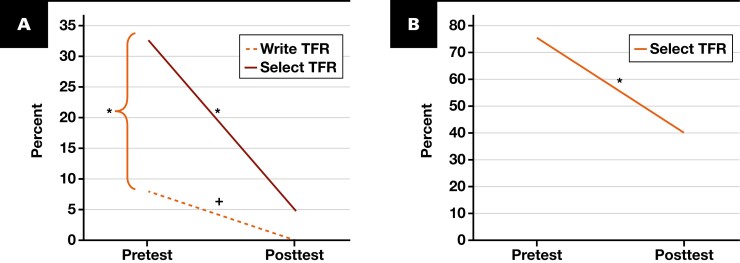
The pre-post evaluation of the transferrin ordering process for the clinician group (**A**) and the nurse group (**B**). *P < .001. + indicates unable to calculate a *P* value because of zero posttest ordering.

As anticipated, the most significant human factor for clinicians and nurses was the confusion between plasma transferrin concentration and plasma transferrin saturation. The educational intervention in the questionnaire aimed to address this root cause in both groups. Analysis of the pretest and posttest data indicated that the educational infographic significantly reduced plasma transferrin ordering in the clinician group for both the writing protocol (from 7.92% to 0%; unable to calculate a *P* value) and the choosing protocol (from 32.7% to 4.95%; *P* < .001) Figure 4A. A similar significant reduction was observed among nurses, from 75.3% to 40% (*P* < .001) Figure 4B.

## DISCUSSION

This study is the first, to our knowledge, to apply RCA to reduce plasma transferrin orders in a clinical laboratory setting. The proposed RCA model encompasses event process description, root cause identification, data collection, significant cause determination, and corrective action implementation Table 1. We identified 22 root causes across 4 main clusters Figure 2. Significant causes were determined through Pareto analysis from questionnaires. For instance, both clinicians and nurses were found to be confused between transferrin concentration and transferrin saturation. Moreover, we applied a concise educational corrective action to our participants, resulting in a significant decrease in transferrin ordering in a postevaluation survey of 84.9% (32.7% to 4.95%, *P* < .001 in choice selecting protocol) in the clinician group and 46.8% (75.3% to 40%, *P* < .001) in the nurse group.

Understanding the root causes of overutilization is essential in LUM. The Clinical and Laboratory Standards Institute recommends that determining the reasons for overutilization is beneficial for developing LUM and serves as the first step before proposing solutions. The institute’s guidelines provide an Ishikawa diagram (fishbone diagram) for a general view of inappropriate test utilization.^1^ Beriault et al^3^ suggest that various quality improvement tools can be used to address overutilization. They recommend both fishbone root cause diagrams and Pareto charts to visualize and evaluate contributing factors. We developed a methodical and easy-to-reproduce model by listing the steps and incorporating quality tools.^21^

In contrast to our work, which applies a systematic RCA model for a specific test overordering, previous studies have relied on authors’ opinions or small group discussions to identify root causes. For instance, a Saudi Arabian study discovered that factors influencing the overutilization of testing included free laboratory services, physicians’ lack of knowledge, patient demand, medicolegal factors, and the multitude of diagnostic tests that are available for doctors to prescribe.^15^ Sedrak et al^23^ reported that among 116 residents from multiple departments, factors contributing to the unnecessary ordering of laboratory investigations in inpatient wards included practice habits, lack of cost transparency, and discomfort with diagnostic uncertainty. Another study by Vrijsen et al^17^ categorized 10 factors of overutilization into 3 major classes (personal, organizational, and technical) and proposed solutions for each cause. Our RCA model offers a detailed understanding of the root causes of testing overuse and facilitates corrective actions empirically demonstrated to reduce it effectively. Therefore, we consider that our comprehensive RCA model is a valuable tool for LUM.

The confusion between laboratory tests emerged as the most reported cause among clinicians and nurses. This issue was also described in a 2020 study by Huang et al.^9^ In addition to transferrin, other tests, such as vitamin D levels, are prone to ordering confusion.^9,24^ The second and third most frequent causes in the clinician group were still related to inadequate knowledge about laboratory tests, as reported in previous studies.^15,17,24,25^ These findings highlight an educational gap related to laboratory testing among clinicians.

Material and machine factors should not be underestimated. Our study discovered that approximately 70% of clinicians and nurses were unaware that the hospital’s website housed the laboratory handbook, which provides information on laboratory tests and their indications. This finding aligns with a recent study by Alshekhabobakr et al,^6^ which reported that 73% of participating doctors did not use the handbook, even though laboratorians made considerable efforts to keep this resource up to date. Struggles with electronic ordering were also found to affect utilization negatively, indicating that information technology can inadvertently hinder laboratory ordering.^6,13^

In method cluster, the system issues represent a significant area for improvement in the laboratory ordering process. Due to the existence of 2 ordering methods (paper based and electronic), we observed that both protocols presented distinct drawbacks. The paper-based protocol, with its multistep collaborative process, increases the risk of misordering by nurses and diminishes the user-friendliness of the ordering process. Furthermore, the paper-based system exhibits a significantly higher overordering rate compared to the single-step electronic orders directly placed by clinicians. Conversely, the electronic ordering system presents challenges related to unclear interface design. This lack of clarity has resulted in confusion among requesters, leading to misordering through accidental clicks.^6,17^

Moreover, the cancellation process for tests and the existence of multiple laboratory systems contributed to the occurrence of ordering errors. Vrijsen et al^17^ identified similar difficult test cancellation in their study, and our institution echoed these findings. This cumbersomeness can lead clinicians to inadvertently retain unnecessary tests ordered through error. In addition, the presence of multiple laboratories and forms also causes a substantial burden for both nurses and clinicians, potentially resulting in further misordering. To effectively address these challenges, supervisors should implement policies promoting proper test selection and consider the simpler laboratory system and cancellation process.

Finally, due to providing the educational infographic, a notable decline in transferrin ordering was found in the pre-post evaluation (a fall of 84.9% in the clinician group). However, residual overordering was still observed at 4.95% and 40% in the clinician and nurse groups, respectively. According to a review by Baird,^12^ education is considered the weakest intervention for LUM. Nonetheless, education is required as a component in certain successful projects and has the lowest risk of unintended negative consequences.^1,3,5,12^ Alternative approaches with potentially stronger impact warrant exploration. A previous study reported using LUM through electronic health record alerts to achieve a 93.4% reduction in transferrin ordering, demonstrating the potential for interventions beyond education.^9^ The additional modalities to reduce redundant transferrin testing are proposed^12^: (1) formulating the iron study test as a single test code excluding plasma transferrin, (2) placing orders on hold for confirmation, (3) stopping payment for this test, and (4) outright banning the test. In accordance with the PDSA cycle, following this study discovery, our laboratory engaged in discussions with key clinicians, including hematologists and pediatricians, regarding the true clinical indications for plasma transferrin. The discussion with our main users reached the agreement that this test was redundant in the presence of the more cost-effective and clinically relevant total iron-binding capacity test. Consequently, the decision to discontinue plasma transferrin as part of our request form was eventually made.

Like other survey-based methodologies, this current study was susceptible to certain types of bias, including recall bias and nonprobability sampling. We designed the questionnaire to evaluate ordering behavior by simulating the investigation of iron deficiency anemia cases in both groups to elucidate recall bias. Moreover, bias from nonprobability sampling was reduced by using multiple sampling modalities: convenience, snowball, and quota. This multimodality approach facilitated the inclusion of a more heterogeneous group of doctors and nurses in the study, thereby expanding the participant number.

Our RCA model intentionally excluded some factors, such as patient demand and medicolegal considerations. These factors were omitted because they are subject to individual clinician judgment and clinical context. In addition, including such factors could potentially undermine clinician decision-making, which is a crucial aspect of high-quality health care delivery. Our identifiable root causes and interventions may not be applicable in every setting due to differences in infrastructure, processes, or other contextual factors.^5^ However, the RCA model we propose can be employed in various situations involving overutilization.

## CONCLUSION

In this research, the RCA model was developed, and its application to LUM was demonstrated. This systematic model enables laboratorians to understand the root causes behind the overuse of their laboratory tests. The educational infographic derived from this model proved effective in correcting transferrin ordering. Although this RCA model is easy to use, the success of its application in improving laboratory utilization depends on how laboratorians can adapt or innovate the model to address unique challenges in different settings.

## Supplementary Material

aqae015_suppl_Supplementary_Material

## Notes

This article is available for CME credit.Go to academic.oup.com/ajcp/pages/journal_cme to see the latest articles. The complete catalog of journal CME courses can be found at store.ascp.org.

## Notes

Conflict of interest disclosure: The authors have nothing to disclose.
