# Supplementary material for: A 5-step root cause analysis model for test overutilization: A study on its application to plasma transferrin testing
Source: Am J Clin Pathol. 2024 Mar 6;162(2):160–6. doi: 10.1093/ajcp/aqae015 (PMC11291954; doi:10.1093/ajcp/aqae015)
Supplement: aqae015_suppl_Supplementary_Material [file aqae015_suppl_supplementary_material.docx]

**Survey: Root Cause Analysis of Plasma Transferrin Ordering in Anemic Patients at Siriraj Hospital**
________________________________________________________________

**For Clinician**

**Part 1: General Information of Respondents** *(Choose options by ticking* ☐*)*

- Position: ☐ Staff ☐ Resident ☐ Fellow ☐ Other : ……………
- Gender: ☐ Male ☐ Female ☐ Not specified
- Department: ☐ Internal Medicine ☐ Pediatrics
   ☐ Obstetrics and Gynecology ☐ Surgery
   ☐ Orthopedic Surgery ☐ Other : ………………………

**Part 2: Information About Ordering Iron Studies**

1. What laboratory tests do you order if iron-deficiency anemia is suspected?

*-* *If you proceed to the next page, you will not be able to go back.-*

1. How often do you complete the test request forms by yourself?
   ☐ Every time ☐ Frequently ☐ Rarely ☐ Never
2. For the tests mentioned in question 1, how do you choose them? (which test and lab)
3.
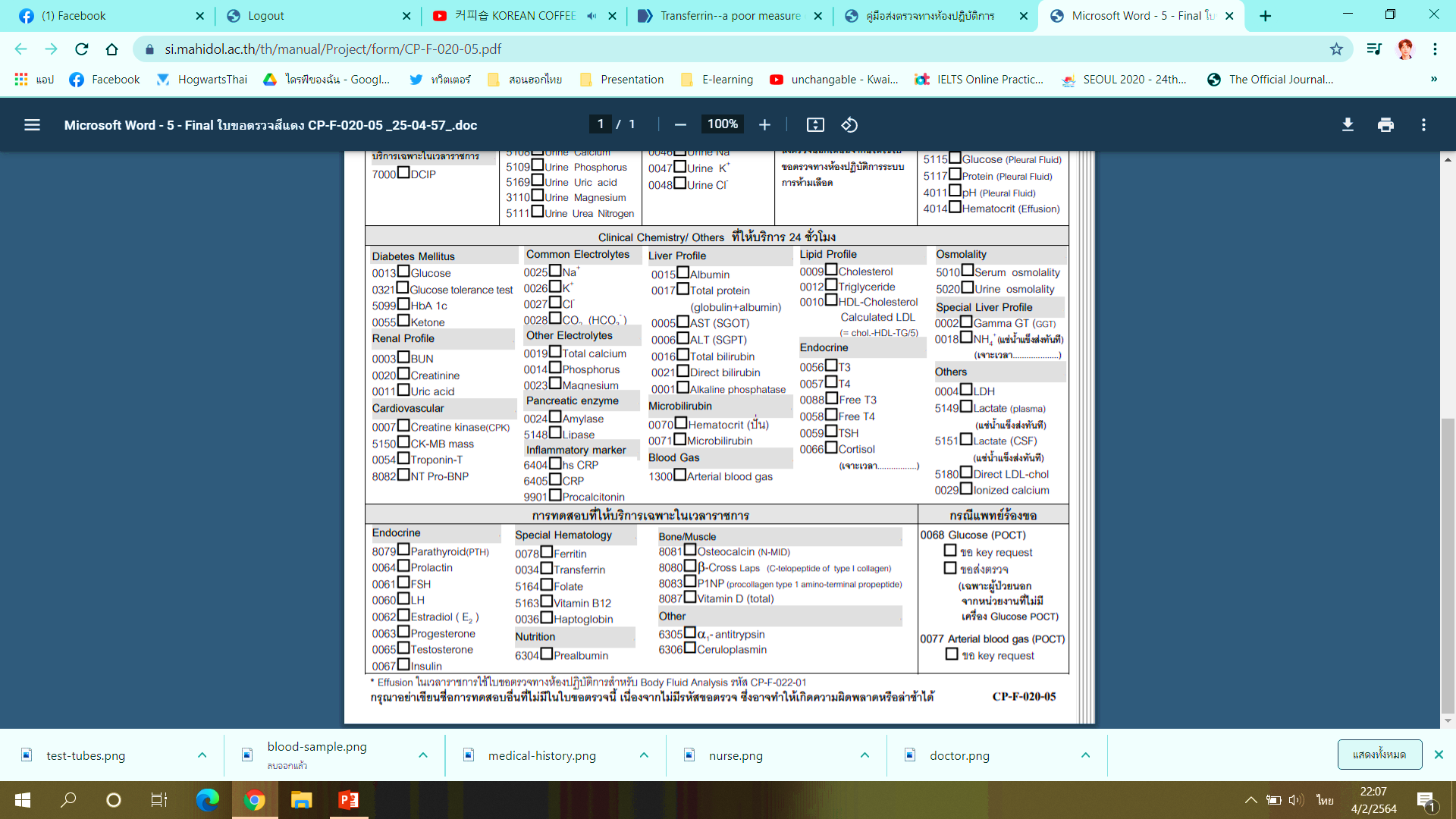
Central Clinical Pathology Laboratory Test Request Form (Select by ticking ☐*)*
4.
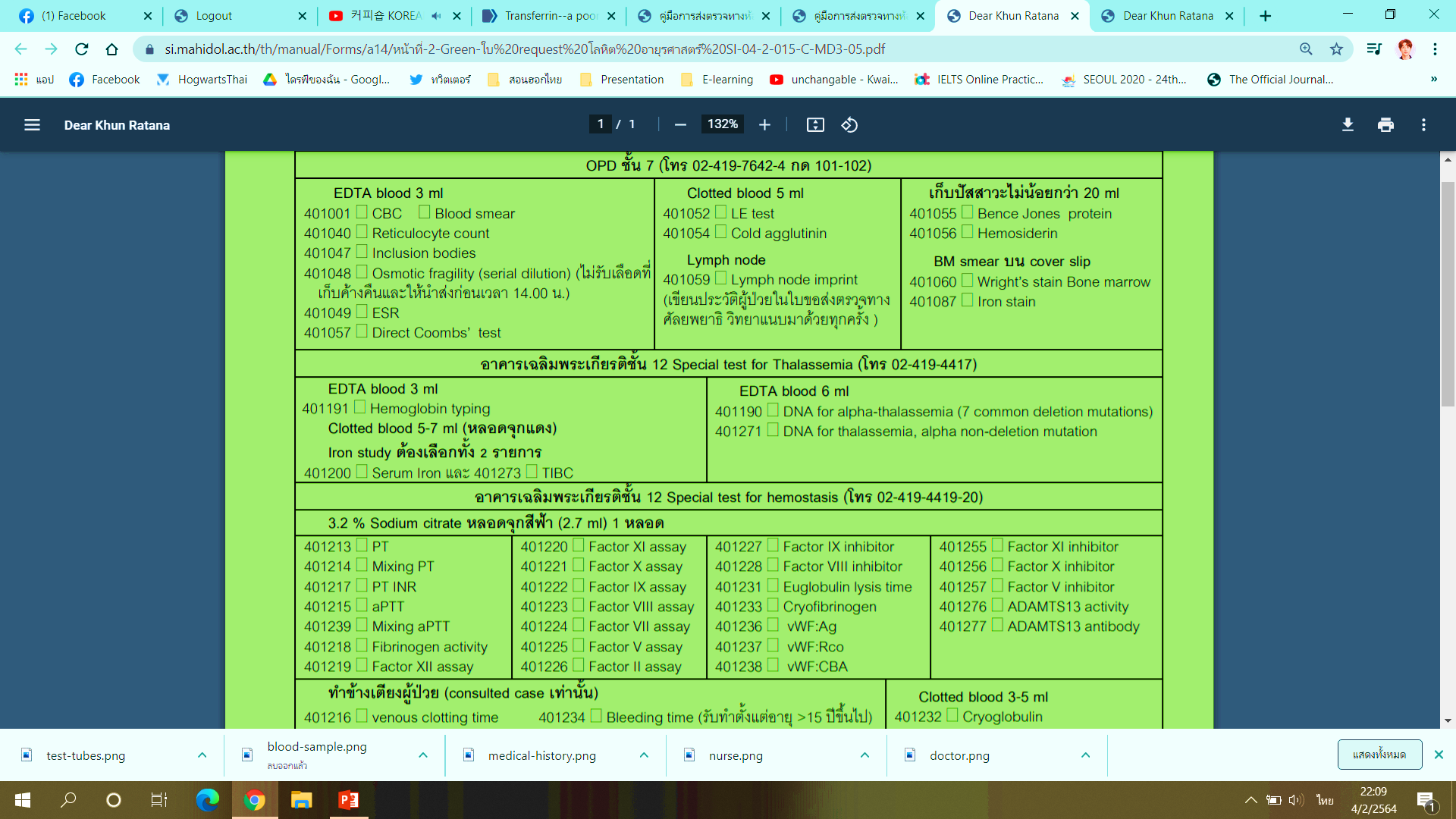
 The Hematology Unit Laboratory Test Request Form (Select by ticking ☐*)*

*-* *If you proceed to the next page, you will not be able to go back.-*


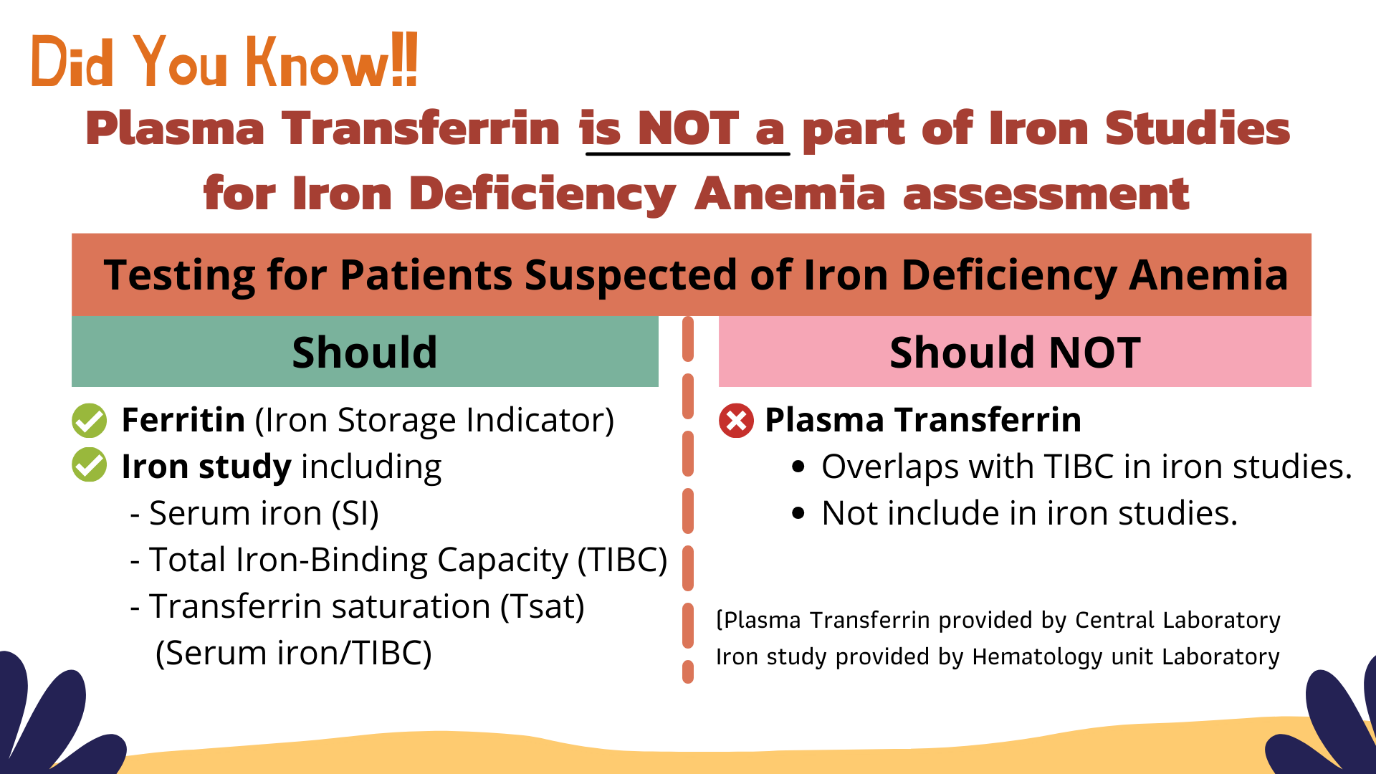


**Part 3: Information About Ordering Iron Studies (Post-intervention test)**

1. What laboratory tests do you order if iron-deficiency anemia is suspected?
2. For the tests mentioned in question 1, how do you choose them? (which test and lab)

Central Clinical Pathology Laboratory Test Request Form (Select by ticking ☐)


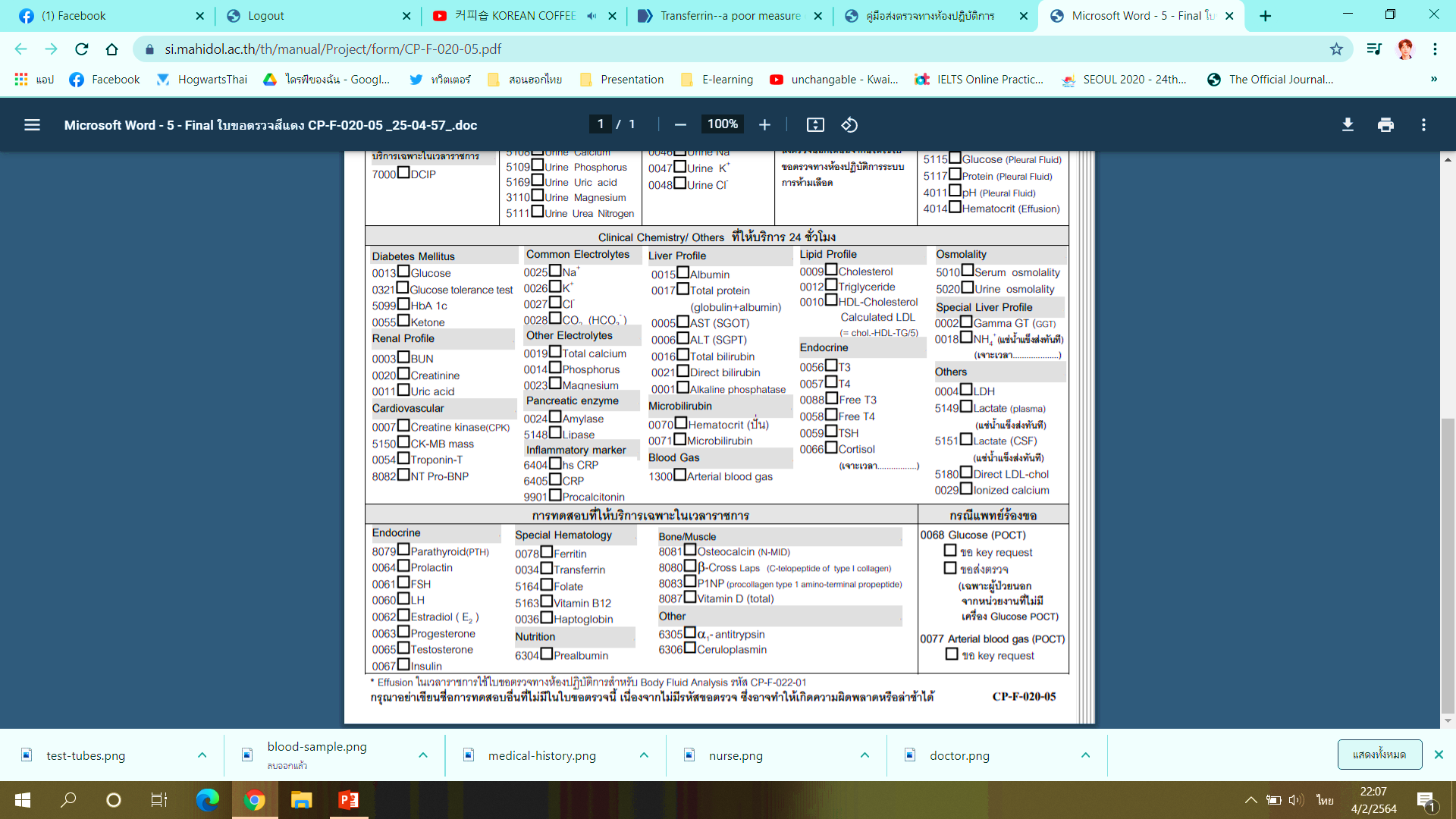


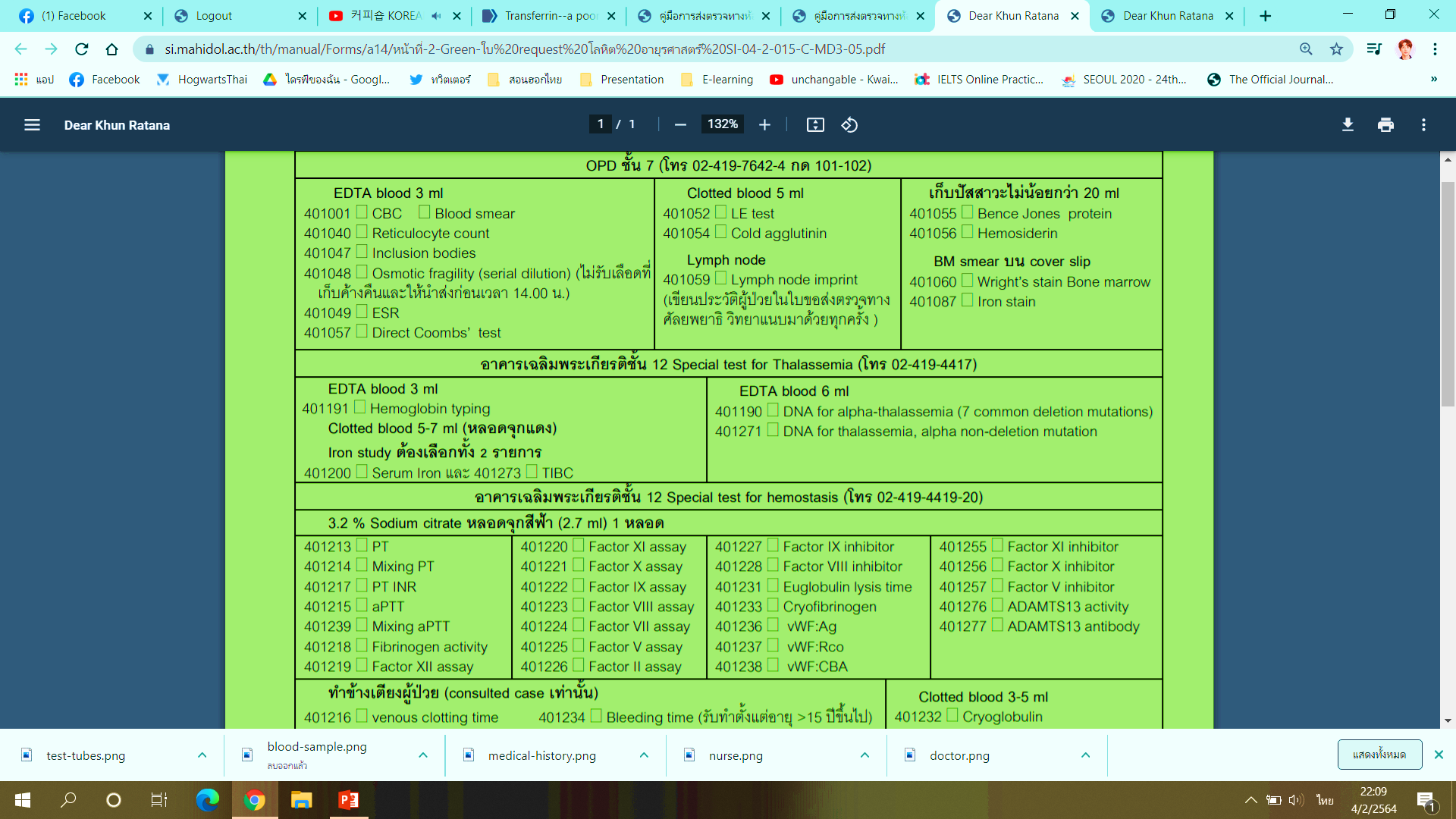
 The Hematology Unit Laboratory Test Request Form (Select by ticking ☐)

**Part 4: Reasons for Ordering Plasma Transferrin Tests in Clinical Practice
(Regarding the tests requested on the Central Clinical Pathology Laboratory)**

| **Considerations** | **Opinion** | |
| --- | --- | --- |
|  | **Yes** | **No** |
| 1. Do you know the difference between plasma transferrin and transferrin saturation? |  |  |
| 1. Do you think plasma transferrin is one of the iron studies that must be ordered? |  |  |
| 1. Do you think plasma transferrin is used in calculating transferrin saturation? |  |  |
| 1. Do you know that plasma transferrin provides results faster than iron studies? |  |  |
| 1. Do you know that plasma transferrin can be used as an alternative for TIBC? |  |  |
| 1. Are you **unsure** about what tests to order, so you order all iron-related tests? |  |  |
| 1. Are you **unaware** of the indications for ordering iron studies and/or transferrin? |  |  |
| 1. Are you **unfamiliar** with providers, request forms, and the method of ordering? |  |  |
| 1. Do you have other reasons for ordering plasma transferrin tests? (Specify).......................................................................................................................................... | (Specify) |  |
| 1. Are you **unfamiliar** with the laboratory handbook for plasma transferrin? |  |  |
| 1. Is the laboratory handbook **unclear**, resulting in confusion in test ordering? |  |  |
| 1. Is the system for in-patient test ordering **difficult**, resulting in erroneous test orders? |  |  |
| 1. Do you think that unnecessary test orders may be due to **other reasons** not related to your clinical need? (Specify).......................................................................................................................................... | (Specify) |  |

**------------ End of survey; Thank you very much --------**

**Survey: Root Cause Analysis of Plasma Transferrin Ordering in Anemic Patients at Siriraj Hospital**

**For Nurse**

**________________________________________________________**

**Part 1: General Information of Respondents** *(Select options by ticking* ☐*)*

- Gender: ☐ Male ☐ Female ☐ Not specified
- Unit: ☐ OPD ☐ IPD ☐ ER (Emergency Room)
- Department: ☐ Internal Medicine ☐ Pediatrics
   ☐ Obstetrics and Gynecology ☐ Surgery
   ☐ Orthopedic Surgery ☐ Other : …………

**Part 2: Entering Iron-related Tests Data**

1. When a physician orders iron-related tests for you to fill the request form, which laboratory service provider do you select for each of the following test? Please select from the following options: *(Select options by ticking* ☐*)*

- Ferritin ☐ Central Laboratory ☐ Hematology Unit (Internal Medicine)
- Transferrin Saturation ☐ Central Laboratory ☐ Hematology Unit (Internal Medicine)
- Serum iron ☐ Central Laboratory ☐ Hematology Unit (Internal Medicine)
- Transferrin ☐ Central Laboratory ☐ Hematology Unit (Internal Medicine)
- TIBC ☐ Central Laboratory ☐ Hematology Unit (Internal Medicine)

1. If the physicians write the following tests in the patient charts for you to request on behalf of them:


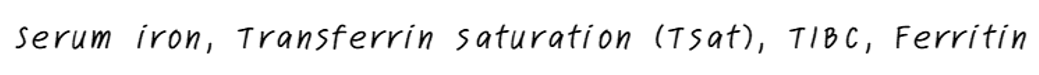


Which request form and which tests will you select?


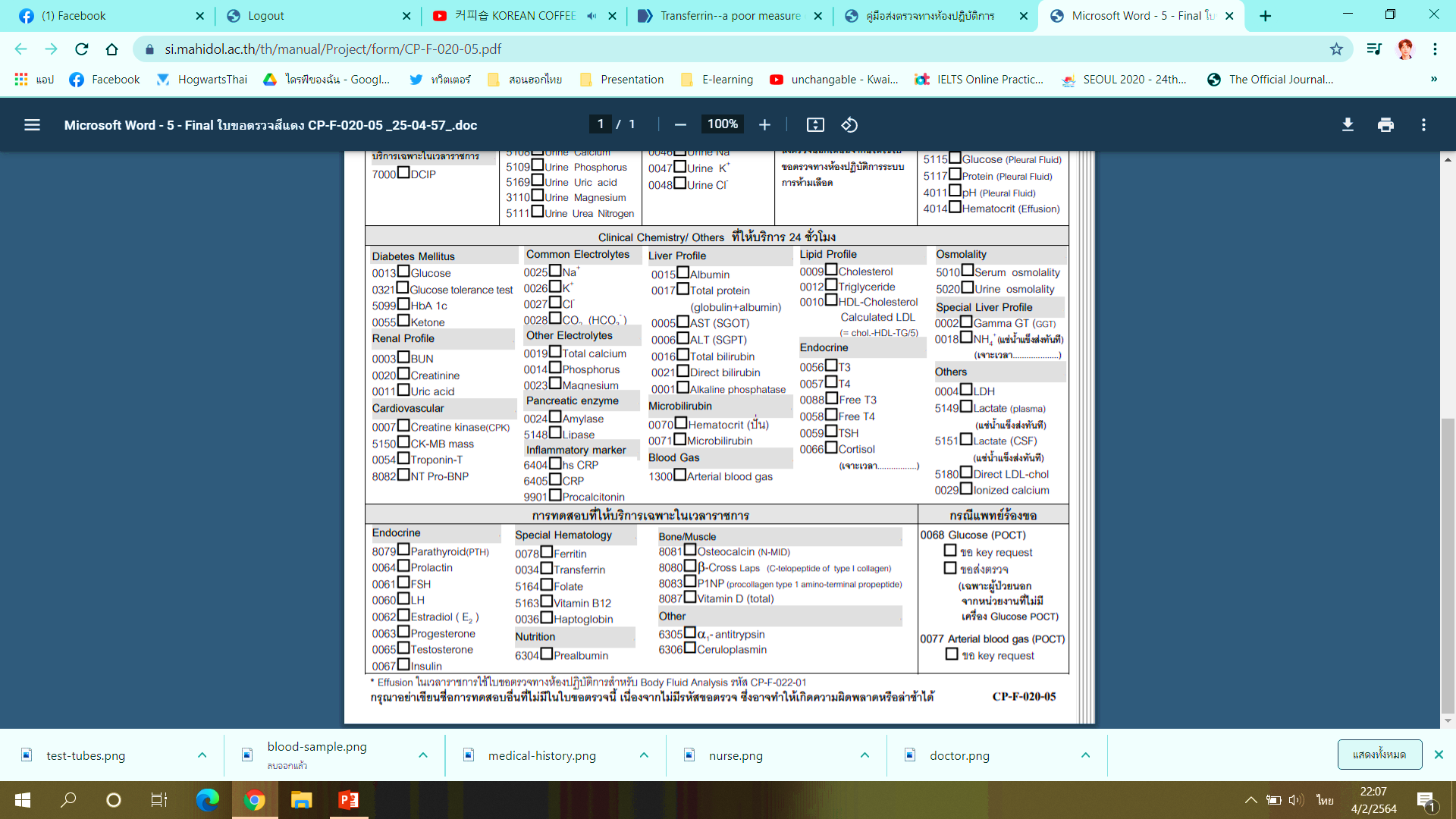
*Form 1: (Select tests as ordered by the physician by ticking* ☐*)*


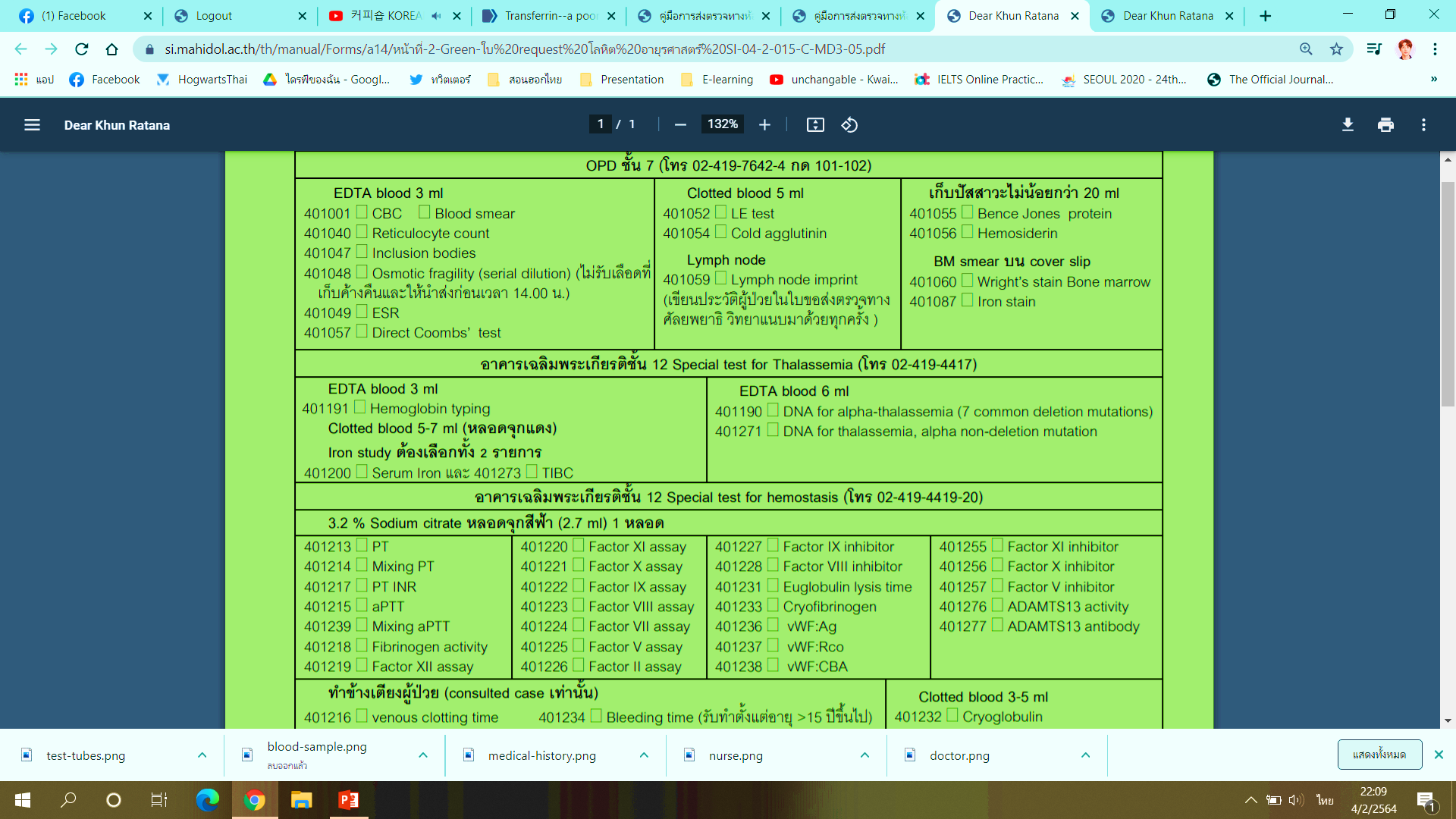

*Form 2: (Select tests as ordered by the physician by ticking* ☐*)*

*.*

*-* *If you proceed to the next page, you will not be able to go back.-*


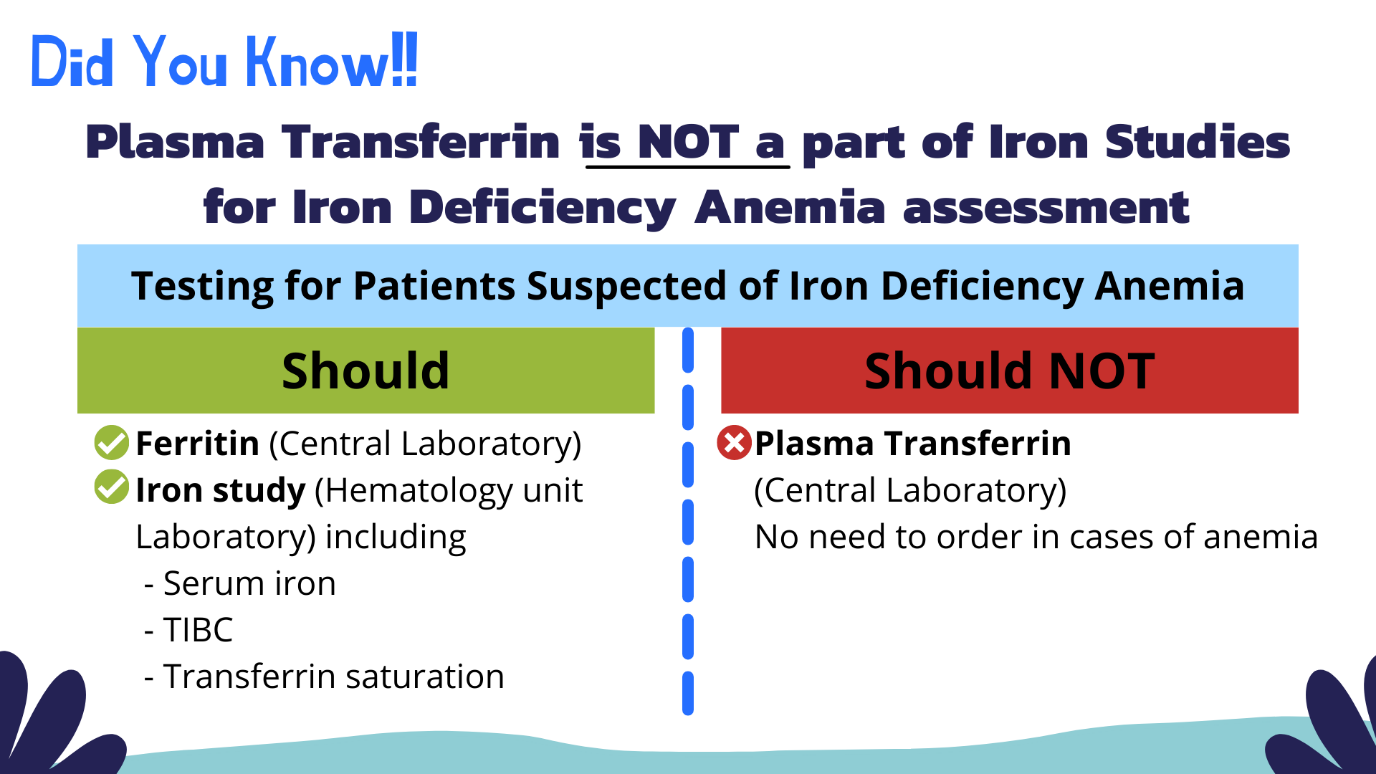


**Part 3: Entering Iron-related Tests Data (Post-intervention test)**

1. If the physicians write the following tests in the patient charts for you to request on behalf of them:


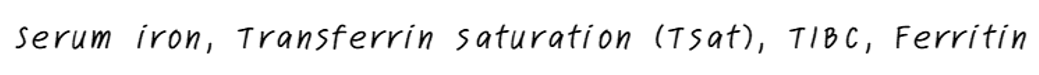


Which request form and which tests will you select?

☐ Central Laboratory
 ☐ Hematology Unit (Internal Medicine)

*Form 1: (Select tests as ordered by the physician by ticking* ☐*)*


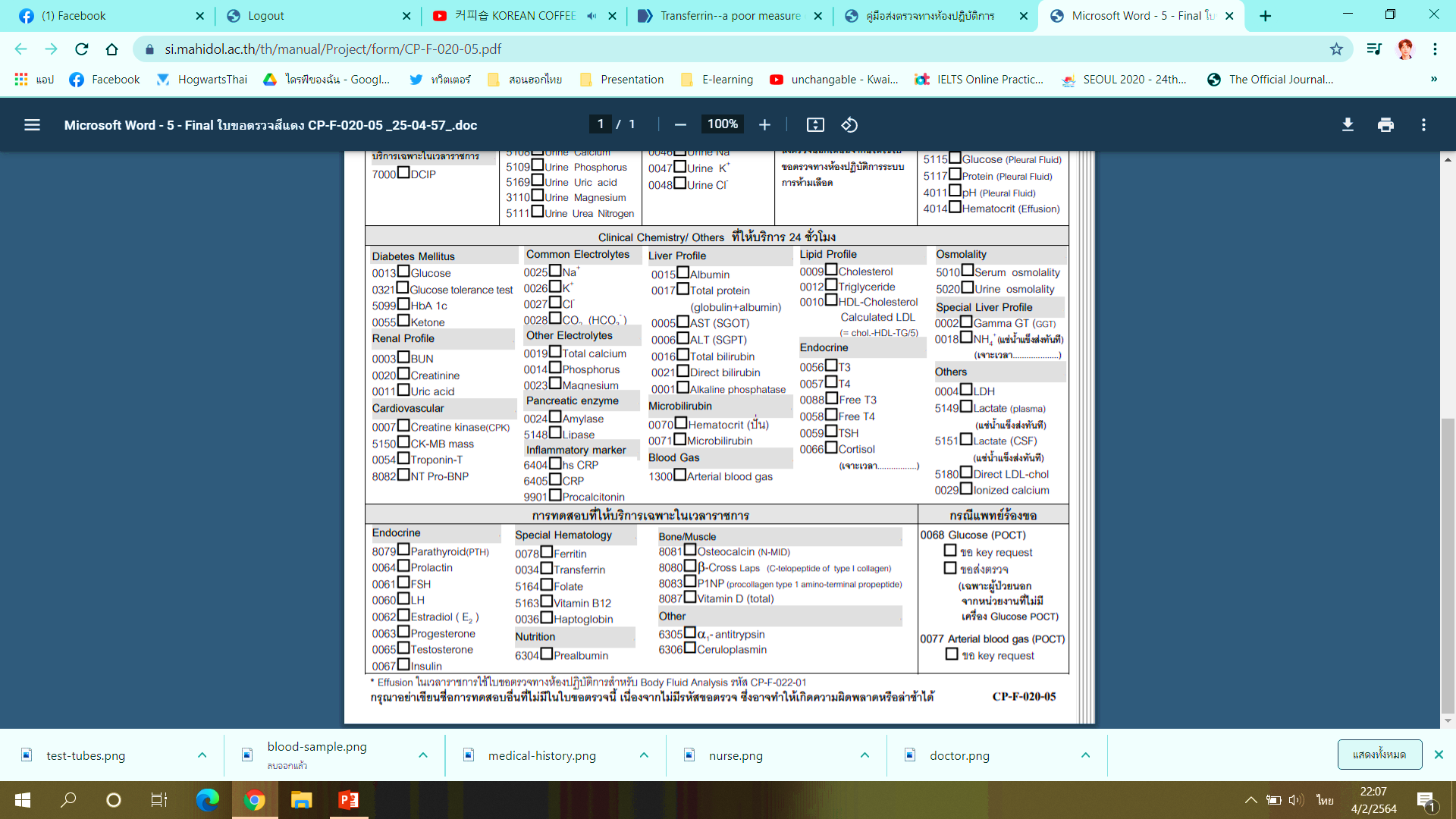


*Form 2: (Select tests as ordered by the physician by ticking* ☐*)*


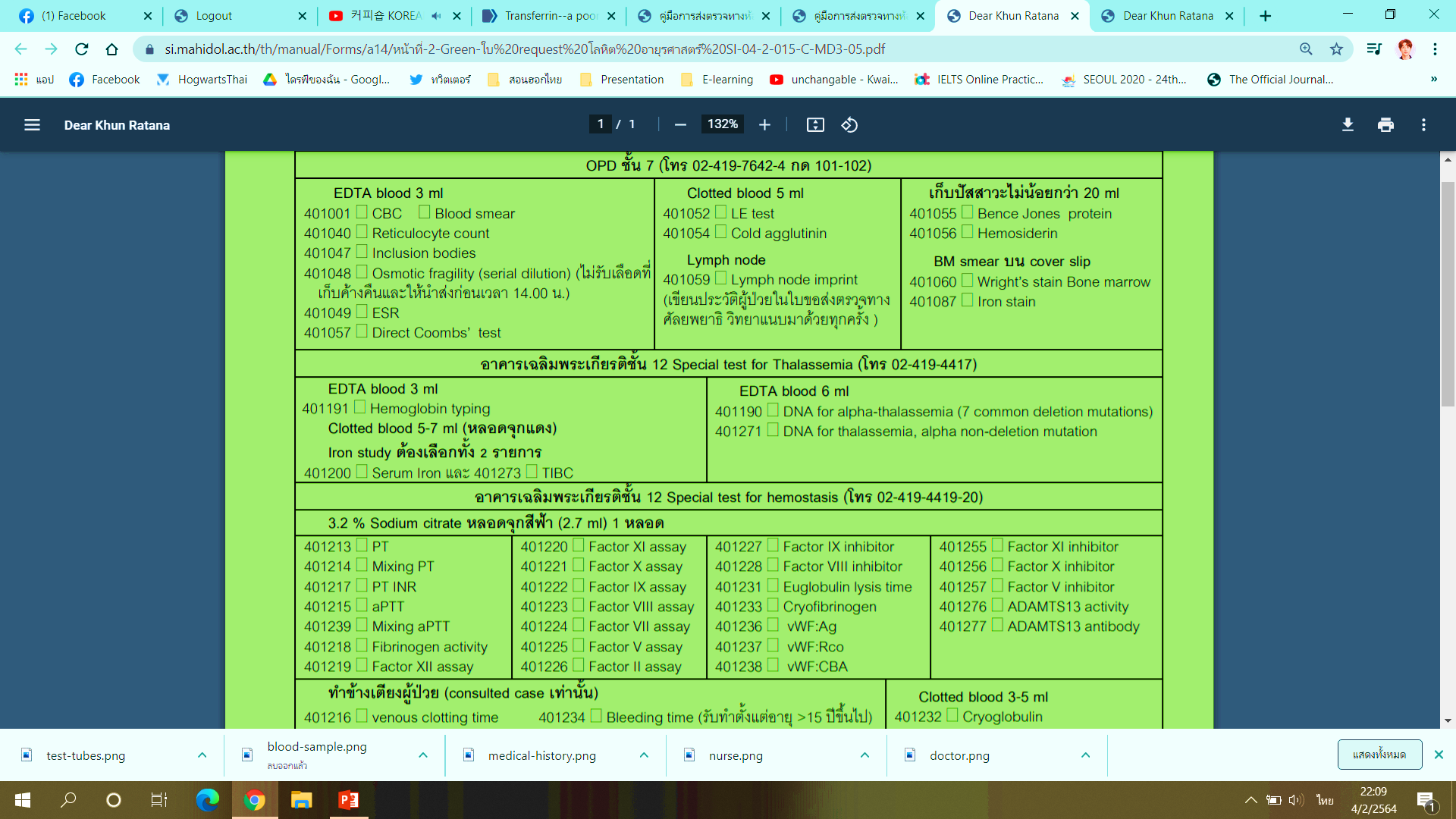


**Part 4: Reasons for Ordering Plasma Transferrin Tests in Clinical Practice**

**(Regarding the tests requested on the Central Clinical Pathology Laboratory)**

| **Considerations** | **Opinion** | |
| --- | --- | --- |
|  | **Yes** | **No** |
| 1. Are you **unaware** of the difference between plasma transferrin and transferrin saturation, leading you to include plasma transferrin in the order? |  |  |
| 1. Do you think that ordering all tests relating to iron including plasma transferrin is necessary to reduce the time for re-venipuncture when if transferrin is needed later? |  |  |
| 1. Are you **unsure** about which test request form to use for iron-related studies? |  |  |
| 1. Do you think it is essential to order plasma transferrin alongside iron studies consistently? |  |  |
| 1. Do you find the test name for plasma transferrin similar to what the physician ordered, leading you to consider ordering it to avoid potential under-testing? |  |  |
| 1. Do you encounter unclear and difficult-to-read physician orders? |  |  |
| 1. Have you noticed that physicians order plasma transferrin in the out-patient charts, and you follow the orders by writing the test request on behalf of them? |  |  |
| 1. Are you **unfamiliar** with the laboratory handbook for plasma transferrin? |  |  |
| 1. Is the laboratory handbook **unclear**, resulting in confusion in test ordering? |  |  |
| 1. Is the in-patient system for test ordering **difficult**, resulting in erroneous test orders? |  |  |
| 1. **Other reason;** (Specify).............................................................................................................. | (Specify) |  |

**----------------- End of survey; Thank you very much ----------------**
